# In search of the altering salivary proteome in metastatic breast and ovarian cancers

**DOI:** 10.1096/fba.2018-00029

**Published:** 2019-01-30

**Authors:** Kuldeep Giri, Anurag Mehta, Kiran Ambatipudi

**Affiliations:** ^1^ Department of Biotechnology Indian Institute of Technology Roorkee Roorkee India; ^2^ Rajiv Gandhi Cancer Institute and Research Centre Delhi India

**Keywords:** neoadjuvant therapy, organotropism, quantitative proteomics, saliva

## Abstract

Breast and ovarian cancers, the most common cancers in women in India, are expected to rise in the next decade. Metastatic organotropism is a nonrandom, predetermined process which represents a more lethal and advanced form of cancer with increased mortality rate. In an attempt to study organotropism, salivary proteins were analyzed by mass spectrometry indicative of pathophysiology of breast and ovarian cancers and were compared to healthy and ovarian chemotherapy subjects. Collectively, 646 proteins were identified, of which 409 proteins were confidently identified across all four groups. Network analysis of upregulated proteins such as coronin‐1A, hepatoma‐derived growth factor, vasodilator‐stimulated phosphoprotein (VASP), and cofilin in breast cancer and proteins like coronin‐1A, destrin, and HSP90α in ovarian cancer were functionally linked and were known to regulate cell proliferation and migration. Additionally, proteins namely VASP, coronin‐1A, stathmin, and suprabasin were confidently identified in ovarian chemotherapy subjects, possibly in response to combined paclitaxel and carboplatin drug therapy to ovarian cancer. Selected representative differentially expressed proteins (eg, gelsolin, VASP) were validated by western blot analysis. Results of this study provide a foundation for future research to better understand the organotropic behavior of breast and ovarian cancers, as well as neoadjuvant drug response in ovarian cancer.

**Abbreviations**
CAB: breast cancer
CAO: ovarian cancer
CAOAC: ovarian cancer after chemotherapy
CORO‐1A: coronin‐1A
GLOBOCAN: global cancer incidence and prevalence
HS: healthy subjects
HSP90α: heat shock protein 90alpha
IDC: invasive ductal carcinomas
SOC: serous ovarian carcinoma
VASP: vasodilator‐stimulated phosphoprotein

## 1 INTRODUCTION

Breast and ovarian cancers are the two most common female malignancies in India, and are ranked fourth and second, respectively, in global rankings of cancer occurrence.^1^ Breast cancer is the leading cause of cancer‐related death in India,^2^ where it metastasizes in distant organs (15%‐30%), or remains within the organ of tumor origin (~10%).^3^ According to a GLOBOCAN report released in 2012, 145 000 new breast cancer cases were diagnosed in India resulting in 70 000 deaths. Together breast and ovarian cancers are responsible for approximately one third of all cancers that occur in women and one fifth of cancer‐related deaths.^4^ Although ovarian cancer is less frequent in India, it is a highly lethal gynecological disease with its incidence steadily rising due to lack of awareness, proper screening protocols, and late detection.^5^ In India, the peak age (age 45‐50 years) of onset of breast and ovarian cancers occurs 10 years earlier, compared to those reported in developed countries.^6^

Cancer metastasis to distant organs and their late‐stage detection are the major cause of mortality in females with breast and ovarian cancers. Metastasis of primary tumors by local invasion into surrounding tissue, and subsequent progression to a distant site where it starts forming new tumor colonies have been observed in many cases, and involve a series of intruding events known as an invasion metastasis cascade.^7^ In breast cancer, invasive ductal carcinoma (IDC), and in ovarian cancer, serous ovarian carcinoma (SOC), are the most frequent and aggressive histological type of cancer metastasizing to different organs.^8^, ^9^ According to the American Cancer Society, the most common type of breast cancer is ductal carcinoma, and lobular carcinoma, which has a 30% probability of spreading to the ovary, is the common type of ovarian cancer.^10^ In addition, depending on the breast cancer molecular subtype, it can spread to different organs such as bones, brain, lungs, and the liver.^8^ Similarly, ovarian cancer has the potential to spread to the abdominal region, and in some cases, to the breast.^11^ However, de novo breast and ovarian cancer metastases, which could depend on the intrinsic properties of both malignant cells and microenvironment of metastasis‐susceptible organ, has also been reported in some cases before the primary diagnosis of cancer.^12^, ^13^ Although breast and ovarian cancers are clinically distinct, mutations in tumor suppressor genes (BRCA1/2, p53, and PTEN) and proto‐oncogenes, and changes in hormone regulation and microenvironment, indicate the similarities between breast and ovarian cancer.^14^ To date, studies have focused on miRNA expression^15^ mutations in breast cancer susceptibility genes BRCA1/2,^16^ and tumor suppressor genes, such as PTEN and P53,^14^ to uncover and predict the metastatic organotropism patterns of breast and ovarian cancers.

Due to the heterogeneous and asymptomatic nature of breast and ovarian cancers, their early detection has been difficult using traditional methods such as mammography,^17^ blood flow patterns by color‐flow Doppler imaging and transvaginal ultrasound examination,^18^ due to high diagnostic costs and radiation exposure. Nevertheless, some degree of success has been achieved for diagnostic and predictive testing using BRCA1/2 mutations^19^ including identification of protein markers for cancer susceptibility using CA15‐3^20^ and CA‐125^21^ from tissue, serum, blood, or saliva, and human epididymis (HE) protein from tissue.^22^ Similarly, mass spectrometry (MS)‐based analysis with two‐dimensional gel electrophoresis (2‐DE) and Affymetrix HG‐U133‐Plus‐2.0 Array have also been extensively used for early detection of different cancers such as lung, gastric, breast, and pancreatic cancers.^23^

Historically, blood has been the primary diagnostic body fluid for analyzing changes in analyte concentration as an indicator of pathophysiological state. However, due to its noninvasive and ease of collection method, saliva has gained momentum as a body fluid that can be used to identify protein markers for a number of diseases, both local (eg, oral, periodontal disease^24^ and primary Sjögren's syndrome^25^) and systemic, including breast and ovarian cancers.^23^ In fact, women with systemic diseases, such as breast and ovarian cancers, have been reported to have impaired salivary gland function and protein composition.^26^ Thus, the aim of the present study was to identify salivary proteins from breast and ovarian cancer patients that could be indicative of metastatic breast and ovarian cancers. Concurrently, an attempt was also made to identify proteins in response to combined drug therapy (paclitaxel and carboplatin). Results of the present study identified a number of differentially expressed salivary proteins (eg, coronin‐1A, vasodilator‐stimulated phosphoprotein) by MS in different groups, in response to drug therapy in post‐chemotherapy ovarian cancer patients. Moreover, this study also provides observations of striking level of interaction among proteins within a group through network analysis.

## 2 MATERIALS AND METHODS

### 2.1 Confirmation of cancer in patients and saliva collection

The clinical research protocol for the collection of human whole saliva, by written informed consent, was approved by the Institutional Review Board (IRB) of Rajiv Gandhi Cancer Institute and Research Centre, Delhi (RGCI & RC). Recruitment of subjects with breast cancer was based on confirmation by mammography, fine needle aspiration cytology (FNAC), and fluorodeoxyglucose‐positron emission tomography (FDG‐PET). Subjects with ovarian cancer were confirmed by FNAC, computed tomography (CT), and FDG‐PET. Effect of chemotherapy was inspected by FDG‐PET.

Saliva samples were collected from healthy human individuals as controls (HS, N = 20), breast cancer patients (CAB, N = 24), ovarian cancer patients (CAO, N = 14), and ovarian cancer patient receiving neoadjuvant chemotherapy (CAOAC, N = 10) with paclitaxel and carboplatin (minimum having/after three cycles of chemotherapy). All females were screened to ensure good oral health with normal salivary function, and were not included in this study if they were undergoing hormonal replacement therapy or lumpectomy or oophorectomy surgery. Unstimulated whole saliva was collected between 5:00 pm to 7:00 pm into sterile falcon tubes and stored on ice. Immediately after collection, 1/20th volume of 1X Protease Cocktail Inhibitor (Roche, Basel, Switzerland) was added to the saliva samples and stored at −80°C until further processing. Clinical details of the CAB, CAO, and CAOAC group patients are listed in Tables 1, 2, 3.

**Table 1:** The key clinicopathological features of breast cancer (CAB) cases used in this study are listed

S. No.	Subject ID	Age/Sex	Ethnicity	Family history	Site of primary detection	Histology	Lymph Node	Pathological/TNM Stage	ER	PR	Her2Neu receptor
1.	CAB1	49/F	Indian	NA	RB	IDC	9/12	IV	70%	Negative	Negative
								pT3N3			
2.	CAB2	65/F	Indian	NA	RB	IDC	11/18	IVC	TNBC		
								pT4N3b			
3.	CAB3	59/F	Indian	NA	LB	IDC	4/16	IVA	70%	60%	10% Positive
								pT3N2a			
4.	CAB4	52/F	Indian	NA	RB	IDC	5/15	IVA	90%	90%	10% Positive
								pT3N2a			
5.	CAB5	61/F	Indian	NA	LB	IDC	NA	IVC	80% Positive	10% Positive	10% Positive
								pT3N2a			
6.	CAB6	49/F	Indian	NA	LB	IDC	4/9	IV	80%	80%	10% Positive
								pT4N3			
7.	CAB7	38/F	Indian	NA	RB	IDC	NA	IVB	TNBC		
								pT4N2			
8.	CAB8	52/F	Indian	NA	LB	IDC	9/16	NA	10%	10% Positive	30% Positive
9.	CAB9	37/F	Indian	NA	LB	IDC	6/27	IVA	70%	70%	30% Positive
								pT4N2			
10.	CAB10	60/F	Indian	NA	LB	IDC	4/10	IVA	NA	NA	NA
								pT3N2a			

CAB, cancer antigen of breast; NA, not applied; RB, right breast; LB, left breast; IDC, invasive ductal carcinomas; ER, estrogen receptor; PR, progesterone receptor; and Her2/Neu, human epidermal growth factor receptor; TNBC, triple negative breast cancer; and pathological stages were classified (TNM staging, T = tissue, N = lymph node, M = metastasis) on the basis of histopathology reports of RGCI&RC.

**Table 2:** The key clinicopathological features of ovarian cancer (CAO) cases used in this study are listed

S. No.	Subject ID	Age/Sex	Ethnicity	Family history	Site of primary detection	Histology/Grade	FIGO Stage	CA125 Value (U/ml)
1.	CAO1	54/F	Indian	NA	Ovary	SC/high grade	IV	4730
2.	CAO2	53/F	Indian	NA	Ovary	SC/high grade	IV	780
3.	CAO3	47/F	Indian	NA	Ovary	SC/high grade	IV	1810
4.	CAO4	41/F	Indian	NA	Ovary	SC/high grade	IIIC	NA
5.	CAO5	54/F	Indian	NA	Ovary	SC/high grade	III	156
6.	CAO6	44/F	Indian	NA	Ovary	SC/high grade	IIIC	NA
7.	CAO7	52/F	Indian	NA	Ovary	SC/high grade	IV	1410
8.	CAO8	45/F	Indian	NA	Ovary	SC/high grade	IIIC	409
9.	CAO9	42/F	Indian	NA	Ovary	SC/high grade	IIIC	677
10.	CAO10	54/F	Indian	NA	Ovary	SC/high grade	IIIC	NA

CAO, cancer antigen of ovary; NA, not applied; SC, serous carcinoma; FIGO, International Federation of Gynaecology and Obstetrics calcification of tumor stage on the basis of histopathology reports of RGCI&RC.

**Table 3:** The key clinicopathological features of ovarian cancer after chemotherapy (CAOAC) cases used in this study are listed

S. No.	Subject ID	Age/Sex	Ethnicity	Family history	Site of primary detection	Histology/Grade	FIGO Stage
1.	CAOAC1	58/F	Indian	NA	Ovary	SC/high grade	IV
2.	CAOAC2	57/F	Indian	NA	Ovary	SC/high grade	IV
3.	CAOAC3	52/F	Indian	NA	Ovary	SC/high grade	IIIC
4.	CAOAC4	60/F	Indian	NA	Ovary	SC/high grade	IIIC
5.	CAOAC5	61/F	Indian	NA	Ovary	SC/high grade	IV
6.	CAOAC6	54/F	Indian	NA	Ovary	SC/high grade	III
7.	CAOAC7	64/F	Indian	NA	Ovary	SC/high grade	IV
8.	CAOAC8	59/F	Indian	NA	Ovary	SC/high grade	IIIC
9.	CAOAC9	63/F	Indian	NA	Ovary	SC/high grade	IV
10.	CAOAC10	55/F	Indian	NA	Ovary	SC/high grade	IV

CAOAC, cancer antigen of ovary after chemotherapy; NA, not applied; SC, serous carcinoma; FIGO, International Federation of Gynaecology and Obstetrics classification of tumor stage on the basis of histopathology reports of RGCI&RC.

### 2.2 Salivary protein extraction

Saliva samples were thawed and centrifuged at 3000× *g* for 15 minutes at 4°C to separate the supernatant (proteins) from the pellet (broken cells). For MS analysis, protein concentration was determined by the Bicinchoninic acid (BCA) protein assay kit (ThermoFisher Scientific, San Jose, CA) as per the manufacturer's instruction. Equal amount of protein (30 µg) was aliquoted from each subject within a group (N = 10) and pooled to normalize the difference between subjects and enhance the detection of low abundant proteins. Twenty‐five micrograms of salivary protein from HS, CAB, CAO, and CAOAC were precipitated with ethanol at 4°C overnight. After centrifugation, the protein pellet was dissolved in 8 M urea/100 mM Tris pH 8.5.

### 2.3 Protein processing and trypsin digestion

The samples were first reduced with 5 mM tris (2‐carboxyethyl) phosphine (TCEP) and cysteines were alkylated by adding 20 mM final concentration of iodoacetamide (IAA). Subsequently, the samples were digested overnight at 37°C in a final concentration of 2 M urea with 100 mM Tris‐HCl, pH 8.5, containing trypsin (Promega, Madison, WI) at an enzyme: substrate ratio of 1:50 for 16 hours at 37°C. The reaction was stopped by addition of 90% formic acid to a final concentration of 4%.^25^ Digested samples were desalted using a C18 silica cartridge (The Nest Group Inc, Southborough, MA).

### 2.4 Protein identification by mass spectrometry

One microgram of the digested peptide mixture were run in technical duplicates on a precolumn and resolved using a 15‐cm Pico‐Frit filled with 1.8 μm C18‐resin in an EASY‐nanoLC 1000 system using an auto sampler (ThermoFisher Scientific). The peptides were eluted using a linear gradient of H2O:CH3CN (98:2, 0.1% formic acid) to H2O:CH3CN (60:40, 0.1% formic acid) at ∼300 nL/min over 105 minutes. High voltage (1800 V) was applied to the low‐volume tee (Upchurch Scientific) and the column tip positioned ∼0.5 cm from the heated capillary of a QExactive mass spectrometer (ThermoFisher Scientific). Positive ions were generated by electrospray with the QExactive operating in top10 HCD data‐dependent acquisition mode with a full scan resolution of 70 000 at m/z 400. MS/MS scans were acquired at a resolution of 17 500 at m/z 400. Lock mass option was enabled for polydimethylcyclosiloxane (PCM) ions (m/z = 445.120025) for internal recalibration during the run.

### 2.5 Database searching

All LC‐MS/MS data were searched using the MASCOT algorithm within Proteome Discoverer 2.2 (ThermoFisher Scientific) against the Uniprot Human proteome database to obtain peptide and protein identifications. For all searches, trypsin was specified as the enzyme for protein cleavage allowing up to two missed cleavages. Oxidation (M) and carbamidomethylation (C) were set as dynamic and fixed modifications, respectively. For Sequest HT and MS Amanda 2.0 search, the precursor and fragment mass tolerances were set at 10 ppm and 0.5 Da, respectively. Both peptide spectrum match and protein false discovery rate were set to 0.01 FDR and determined using percolator node. Relative protein quantification of the proteins was performed using the Minora feature detector node of Proteome Discoverer2.2 with default settings using peptide spectrum matches (PSM) with confidence. The MS data has been deposited to the ProteomeXchange Consortium (http://proteomecentral.proteomexchange.org) with the PRIDE partner repository with the dataset identifier PXD011541.

Search Tool for the Retrieval of Interacting Genes (STRING) version 10.5 (http://string-db.org/), an online protein‐protein interaction database, curated from literature and predicted associations from systemic genome comparisons was used to identify protein‐protein interactions. Proteins from all four groups were analyzed and their interactions and functional linkage was displayed according to their confidence, evidence, actions, or interactions.

### 2.6 Statistical analysis

The statistical analysis of the dataset containing up‐ or downregulated proteins (arbitrary fold change cut‐off ≥2 or ≤0.5) in all four samples including proteins identified only in one group (eg, HS, CAB, CAO, and CAOAC) were analyzed by Origin Software, 2018 (Origin Lab Corporation, http://www.OriginLab.com, Northampton, MA) and Microsoft Office Excel 2013. To compare all four sample groups, one‐way ANOVA (*P* < 0.05) was applied. Heat maps were generated by hierarchical clustering with Euclidean distance and average linkage by using the Heat mapper webserver.^27^ The list of all proteins found in HS, CAB, CAO, and CAOAC were annotated and summarized at different gene ontology categories using the PANTHER Classification System, 13.1.^28^ Venn diagrams across four sample sets were generated in Origin software. A paired *t*‐test was applied for each comparison to generate volcano plots using Origin software.

### 2.7 Western blot

The differential expressions of selected proteins were confirmed in pooled samples (N = 10) across four groups (HS, CAB, CAO, and CAOAC). Additionally, protein expression was verified on individual subjects/patients using two cohorts to determine if an outlier was influencing our results: cohort 1: subjects/patients (N = 5) used for the discovery dataset and cohort 2: subjects/patient (N = 5), an independent cohort. Forty micrograms of protein from each group was separated in 12% SDS PAGE in a Mini‐PROTEAN® Tetra Hand cast System (Bio‐Rad, Hercules, CA). Protein was transferred onto nitrocellulose membranes (Pall Corporation, Pensacola, FL) for 2 hours at 30 V. Membranes were blocked for 2 hours with 5% nonfat dry milk with 0.05% Tween 20 (TBST) at room temperature. Subsequently, blots were probed with antibodies against rabbit polyclonal fibrinogen‐α (1:1000, Santracruz, TX) as a loading control, rabbit polyclonal gelsolin (1:1000, Sigma Aldrich, St. Louis, MO), rabbit monoclonal glyceraldehyde‐3‐phosphate dehydrogenase (ThermoFisher Scientific), rabbit monoclonal vasodilator‐stimulated phosphoprotein (1:500, Cell signaling technology, Danvers, MA), or rabbit polyclonal haptoglobin (1:1000, Elabscience, Wuhan, China). The secondary antibodies were horse radish peroxidase (HRP)‐labeled goat anti‐rabbit IgG (Santracruz, Dallas, TX) or goat anti‐mouse IgG (Santracruz), incubated at room temperature for 1 hour, developed using ECL Western blotting detection reagents (ThermoFisher, Scientific) and visualized using the ChemiDoc Imaging System (Bio‐Rad).

## 3 RESULTS

### 3.1 Screening of tumor by imaging

**Figure 1:**
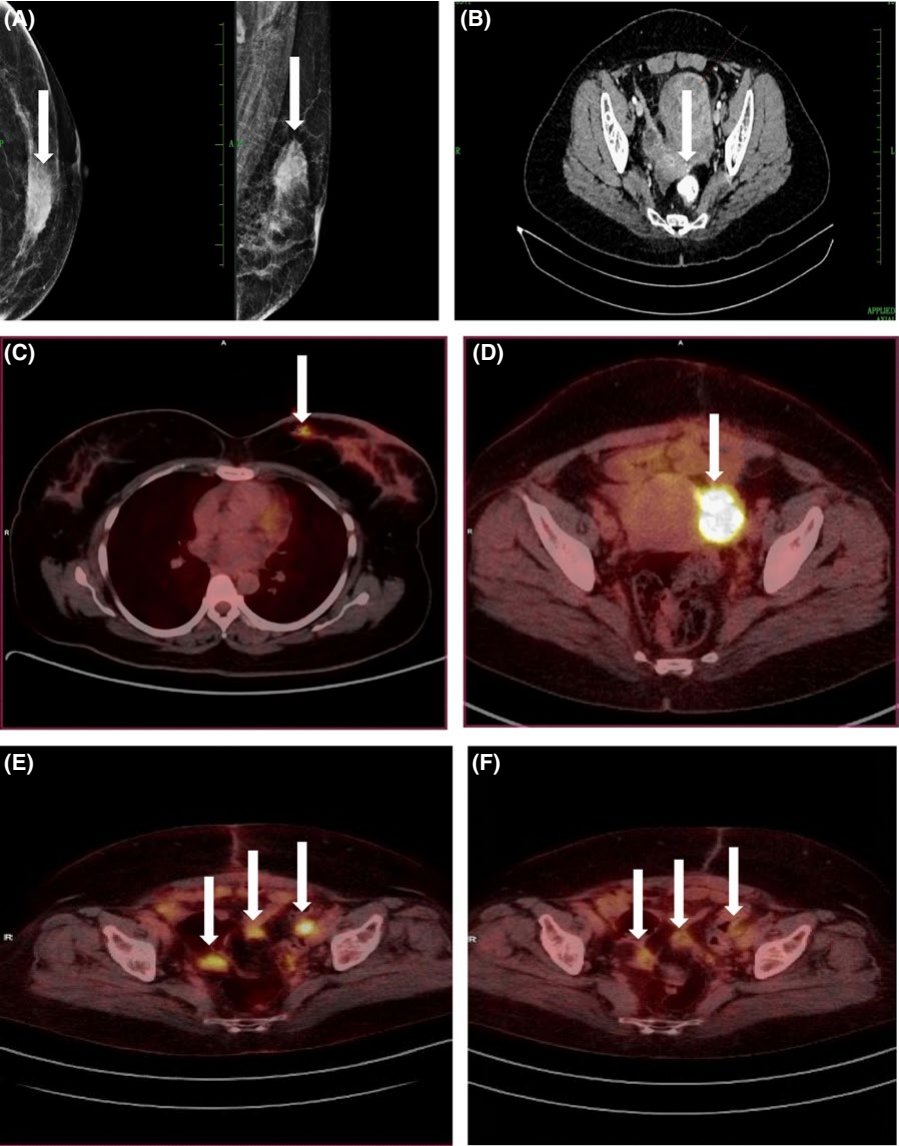
Screening of breast and ovarian tumors by imaging. (A) Left image: mammogram of left breast lesion indicated by an arrow. (B) CT scan of ovarian lesion indicated by an arrow. (C and D) FDG‐PET/CT image of breast and ovarian lesions indicated by an arrow, respectively. (E) FDG‐PET/CT image of ovarian tumor before neoadjuvant paclitaxel and carboplatin drug therapy, and (F) tumor size reduction after three cycles of combined drug therapy with lesions indicated by arrows before and after therapy

The primary screening of breast and ovarian lesions of unknown primary site in women was performed by mammography and CT scans, respectively. Lesions in the left breast (indicated by an arrow in a representative case) show high density with irregular margins in the upper inner quadrant in middle depth (Figure 1A). Based on the Breast Imaging‐Reporting and Data System (BIRADS), the lesion was categorized as a five and positive for a predictive chance (approximately 95%) of having breast cancer. Ovarian lesions observed through CT scan (indicated by an arrow in a representative case) were diagnosed as a bilateral adnexal solid cystic lesion (Figure 1B). The left adnexal lesion measured 6.9 × 6.4 cm, while the right adnexal lesion was 2.7 × 2.4 cm with heterogeneous pattern of enhancement. The clinical findings by CT scan indicate a case of ovarian cancer with elevated levels of serum CA125: 81 U/mL (Normal CA125: <35 U/mL).

Primary screening performed using FDG‐PET/CT, based on the standardized uptake values of glucose (Normal: <2.5), to determine the lesion as malignant or normal showed a score of 5.8 and 19.2 for breast and ovarian lesions, respectively, indicating malignancy. The FDG‐PET/CT images for breast and ovarian lesions are indicated by arrows including their SUVs (Figure 1C,D). Furthermore, FDG‐PET/CT performed to predict the effect of neoadjuvant chemotherapy on the size of ovarian tumors showed 9.6 prior to chemotherapy (Figure 1E), while it was 3.1 after three cycles of combinational drug treatment (Figure 1F).

### 3.2 Diagnosis of subjects with tumor by histopathology

Whole saliva samples were collected from subjects confirmed with either breast or ovarian tumor by histopathology, including confirmation of hormone receptor (HR) status (ER/PR and HER2/neu) in breast cancer subjects using immunohistochemistry (IHC).

All the breast cancer patient (N = 10) tumors included in this study were histologically defined as invasive ductal carcinoma (IDC) subtypes and clinical/pathological stage IV. Two patients had triple negative breast cancer (ER‐, PR‐, and Her‐) and all other subjects were either ER/PR positive/negative or Her2 positive/negative. HR status or immunohistochemistry was performed to determine the aggressiveness of breast cancer, as the expression of Her2/neu positive (luminal B and Her‐2 type) receptors is known to indicate an increase in aggressiveness of breast cancer or metastasis of breast cancer (MBC), while triple negative breast cancer grows very fast with poor prognosis.^29^ Similarly, higher expression levels of progesterone positive receptor (Her‐2 overexpression) in breast cancers has been correlated with later stage and promote tumor growth in younger women.^30^ All the ovarian cancer subjects (N = 10) and patients under treatment with neoadjuvant drug (N = 10) had tumors that histologically were defined as stage IV serous ovarian carcinomas. The key clinicopathological features of breast and ovarian cancer cases used in this study are listed in Tables 1, 2, 3.

### 3.3 Overall comparison of differentially expressed proteomic profiles between HS, CAB, CAO, and CAOAC

**Figure 2:**
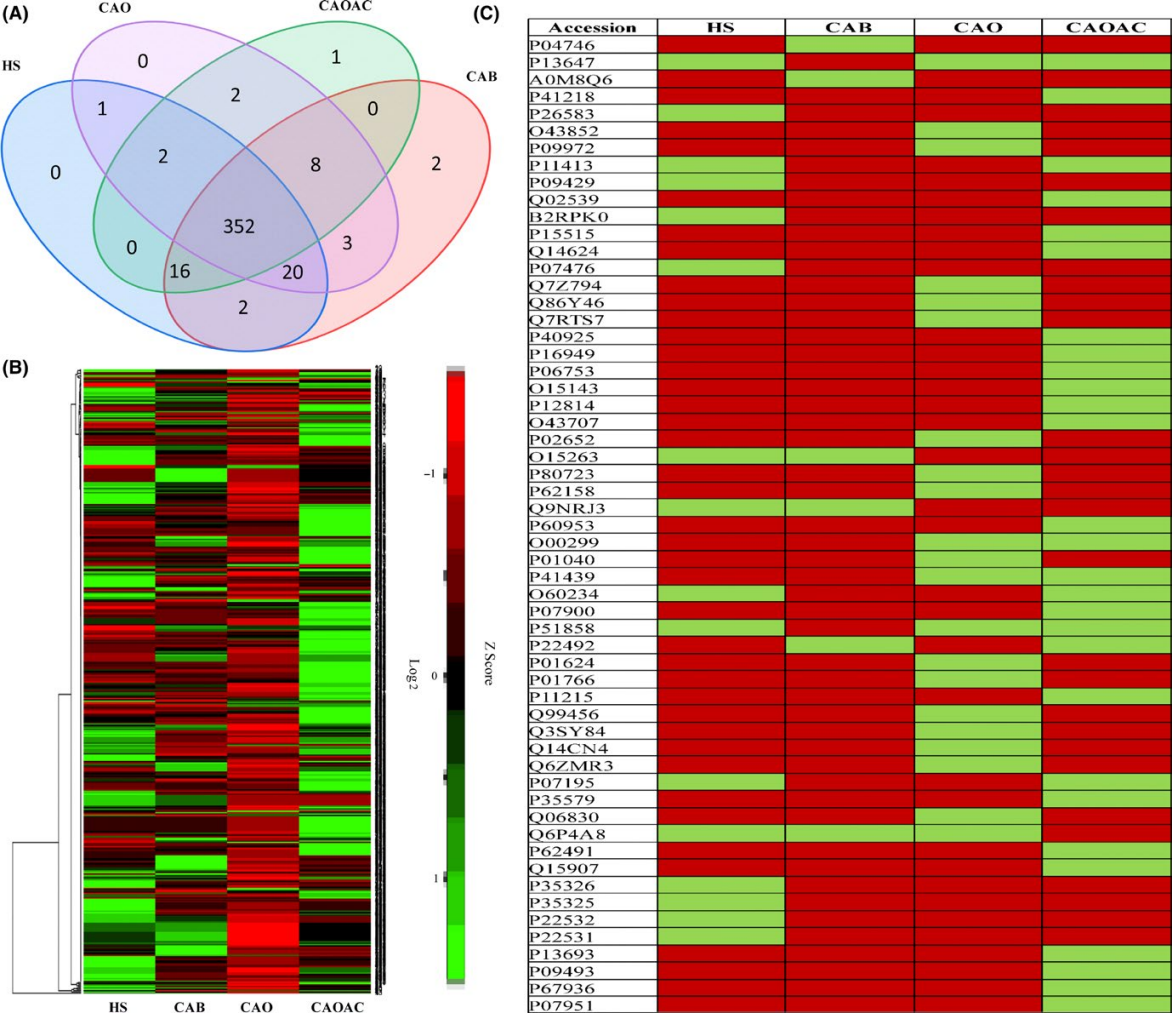
Differential expression of proteins in four groups by using proteins’ Uniprot IDs. (A) Venn diagram showing the comparison of the proteins, (B) hierarchical cluster analysis of differentially expressed proteins across healthy subjects (HS), breast cancer (CAB), ovarian cancer (CAO), and ovarian cancer subjects after chemotherapy (CAOAC) groups, and (C) heat map representing proteins which are either present (red) or absent (green) exclusively in one group or common in any two/three groups

Differential expression of proteins was detected among HS, CAB, CAO, and CAOAC subjects. Each protein was deemed a confident match when the detection of at least two unique/different peptides was observed from a combined pool of MS/MS. Multiple analyses were performed for each pool by label‐free quantification, and resulted in the identification of 645 proteins, of which 409 proteins were confidently identified and were subsequently analyzed. Collectively, a curated protein list from the four groups was compiled and a quantitation of alterations in abundance was performed based on precursor ion intensity. A Venn diagram (Figure 2A) showed 352 common proteins and hierarchical clustering (Figure 2B) showed the pattern of change in protein abundance across all groups. Additionally, another heat map was generated to show if a protein was present (red) or absent (green) (Figure 2C) and to this end a total of 57 differentially expressed proteins were either present/absent exclusively in one group or common in any two/three groups.

### 3.4 Group‐wise comparison of identified proteins

A group‐wise comparison of proteins identified by the MS/MS performed against Uniprot Human database showed proteins either with no change in abundance (common in each group) or differentially expressed in each group including proteins identified only in one group. Supplementary Table S1.

#### 3.4.1 Expression of proteins in CAB compared to HS

**Figure 3:**
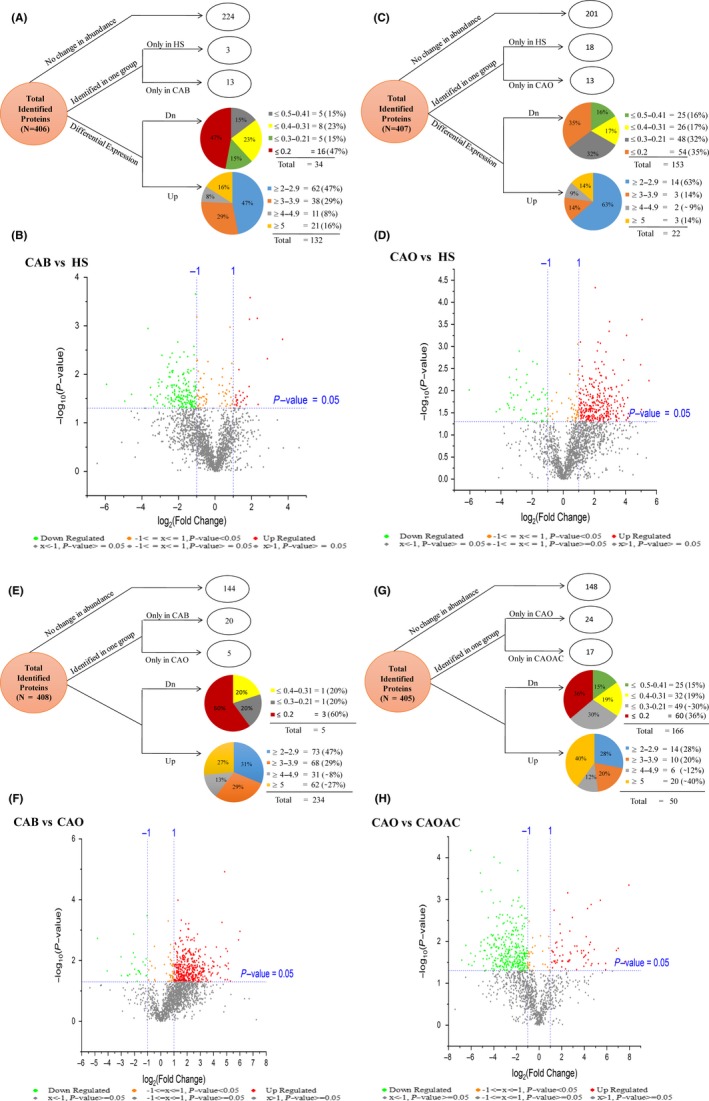
Distribution of differentially expressed proteins detected by mass spectrometry using an arbitrary fold ratio change cut‐off of ≤0.5 or ≥2. (A) In breast cancer (CAB) and healthy subjects (HS) (N = 406), proteins were categorized into proteins that did not change in abundance (N = 224), proteins identified only in healthy subjects (HS) (N = 3), proteins identified only in CAB (N = 13), and proteins downregulated in CAB (N = 34) and upregulated in CAB (N = 132). (B) Graphical representation of the significance of the differential expression in CAB and HS, quantitatively was performed using volcano plots—log_10_ (*P* value) vs log_2 _(fold change). (C) Proteins detected by mass spectrometry in HS and ovarian cancer (CAO) (N = 407) were categorized into proteins that did not change in abundance (N = 201), proteins identified only in HS (N = 18), proteins identified only in CAO (N = 13), and proteins downregulated in CAO (N = 153) and upregulated in CAO (N = 22). (D) Graphical representation of the significance in the differential expression in CAO and healthy subjects (HS), quantitatively was performed using volcano plots—log_10_ (*P* value) vs log_2 _(fold change). (E) Proteins detected by mass spectrometry in CAB and CAO (N = 408) were categorized into proteins that did not change in abundance (N = 144), proteins identified only in CAB (N = 20), proteins identified only in CAO (N = 5), and proteins downregulated in CAO (N = 5) and upregulated in CAO (N = 234). F). Graphical representation of the significance in the differential expression in CAB and CAO, quantitatively was performed using volcano plots—log_10_ (*P* value) vs log_2 _(fold change). (G) Proteins detected by mass spectrometry in CAO and ovarian cancer subjects after chemotherapy (CAOAC) (N = 405) were categorized into proteins that did not change in abundance (N = 148), proteins identified only in CAO (N = 24), proteins identified only in CAO subjects after CAOAC (N = 17), and proteins downregulated in CAO (N = 166) and upregulated in CAO (N = 50), and (H) graphical representation of the significance in the differential expression in CAO and ovarian cancer subjects after CAOAC, quantitatively was performed using volcano plots—log_10_ (*P* value) vs log_2 _(fold change)

A total of 406 proteins were collectively identified in saliva samples from CAB patients and HS with less than 1% FDR. Based on abundance ratio (≥2 or ≤0.5) of normalized precursor ion intensity, 166 proteins were differentially expressed, of which 132 proteins were upregulated, while 34 were downregulated including 224 showing no change in abundance in CAB compared to HS. In addition, 13 and 3 proteins were identified exclusively in CAB and HS, respectively (Figure 3A). Graphical representation of the significance in the differential expression was quantitatively performed using volcano plots—log_10_ (*P* value) vs log_2 _(fold change) (Figure 3B).

#### 3.4.2 Expression of proteins in CAO compared to HS

A total of 407 proteins were collectively identified in saliva samples from CAO patients and HS with less than 1% FDR. Based on abundance ratio (≥2 or ≤0.5) of normalized precursor ion intensity, 175 proteins were differentially expressed, of which 22 were upregulated while 153 were downregulated including 201 showing no change in abundance in CAO patients as compared to HS. In addition, 13 and 18 proteins were identified exclusively in CAO patients and HS, respectively (Figure 3C). Graphical representation of the significance in the differential expression was quantitatively performed using volcano plots—log_10_ (*P* value) vs log_2 _(fold change) (Figure 3D).

#### 3.4.3 Expression of proteins in CAB compared to CAO

A total of 408 proteins were collectively identified in saliva samples from CAB and CAO patients with less than 1% FDR. Based on abundance ratio (≥2 or ≤0.5) of normalized precursor ion intensity, 239 proteins were differentially expressed, of which 234 were upregulated while 5 were downregulated including 144 showing no change in abundance in CAB as compared to CAO patients. In addition, 20 and 5 proteins were identified exclusively in CAB and CAO, respectively (Figure 3E). Graphical representation of the significance in the differential expression was quantitatively performed using volcano plots—log_10_ (*P* value) vs log_2 _(fold change) (Figure 3F).

#### 3.4.4 Expression of proteins in CAO compared to CAOAC

A total of 405 proteins were collectively identified in saliva samples from CAO and CAOAC patients with less than 1% FDR. Based on abundance ratio (≥2 or ≤0.5) of normalized precursor ion intensity, 216 proteins were differentially expressed, of which 50 were upregulated while 166 were downregulated including 148 showing no change in abundance in CAO as compared to CAOAC patients. In addition, 24 and 17 proteins were found exclusively in CAO and CAOAC, respectively (Figure 3G). Graphical representation of the significance in the differential expression was quantitatively performed using volcano plots—log_10_ (*P* value) vs log_2 _(fold change) (Figure 3H).

### 3.5 Functional classification of identified proteins

**Figure 4:**
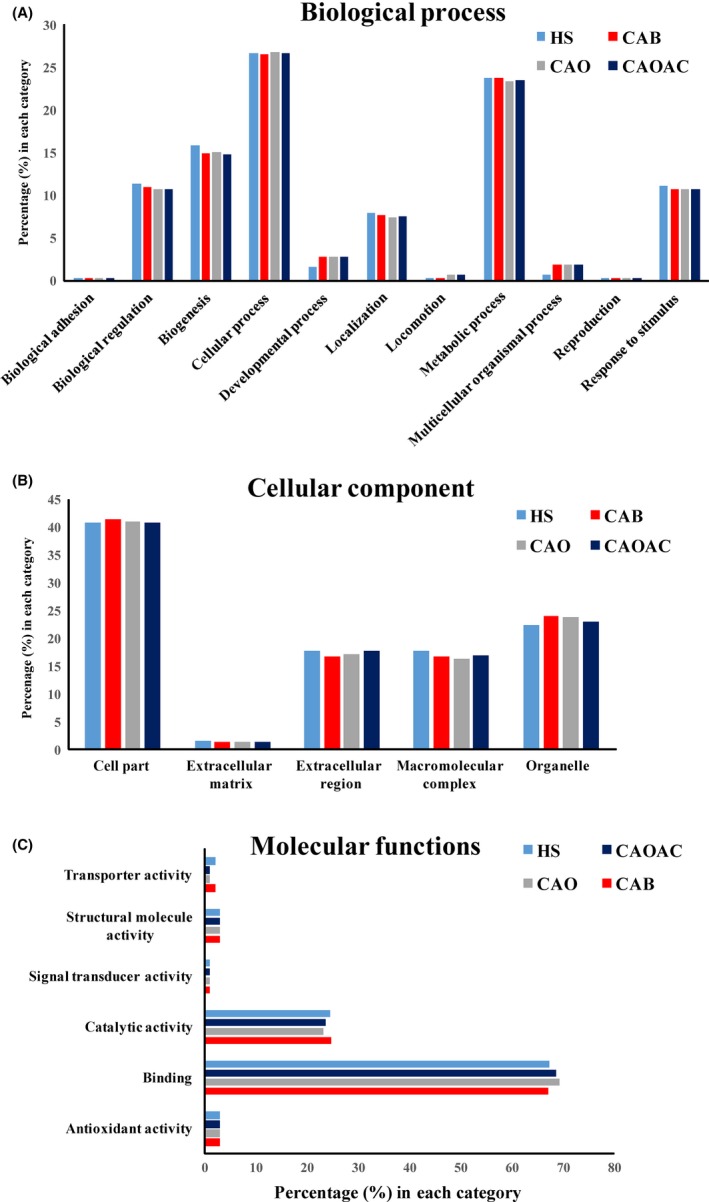
Characterization of human salivary proteins by GO analysis. Functional distribution of the 433 proteins identified proteins with mass spectrometry from healthy subjects (HS), breast cancer (CAB), ovarian cancer (CAO), and ovarian cancer subjects after chemotherapy (CAOAC) patient groups in accordance with (A) biological function (BF), (B) cellular component (CC), and (C) molecular function (MF)

All proteins with their respective Ensembl Gene IDs were characterized by gene ontology (GO) using PANTHER Classification System 13.1. Based on the biological processes, the proteins were classified into those involved in cellular processes (27%), metabolism (24%), cellular component organization or biogenesis (15%), response to stimulus (11.42%), biological regulation (11.11%), localization (8%), and developmental process (1.5%) (Figure 4A). The classification based on cellular components revealed the majority of the proteins were involved in the formation of cellular parts (41%), followed by organelle fractions (24%), extracellular (16%), or the macromolecular complex (16%) (Figure 4B). Analysis based on molecular function showed that most of the proteins were involved in catalytic activity (42%) and binding (40%), followed by structural molecule activity (3%), antioxidant activity (3%), transporter activity (2%), and signal transducers (1%) (Figure 4C).

### 3.6 Protein interaction network analysis

**Figure 5:**
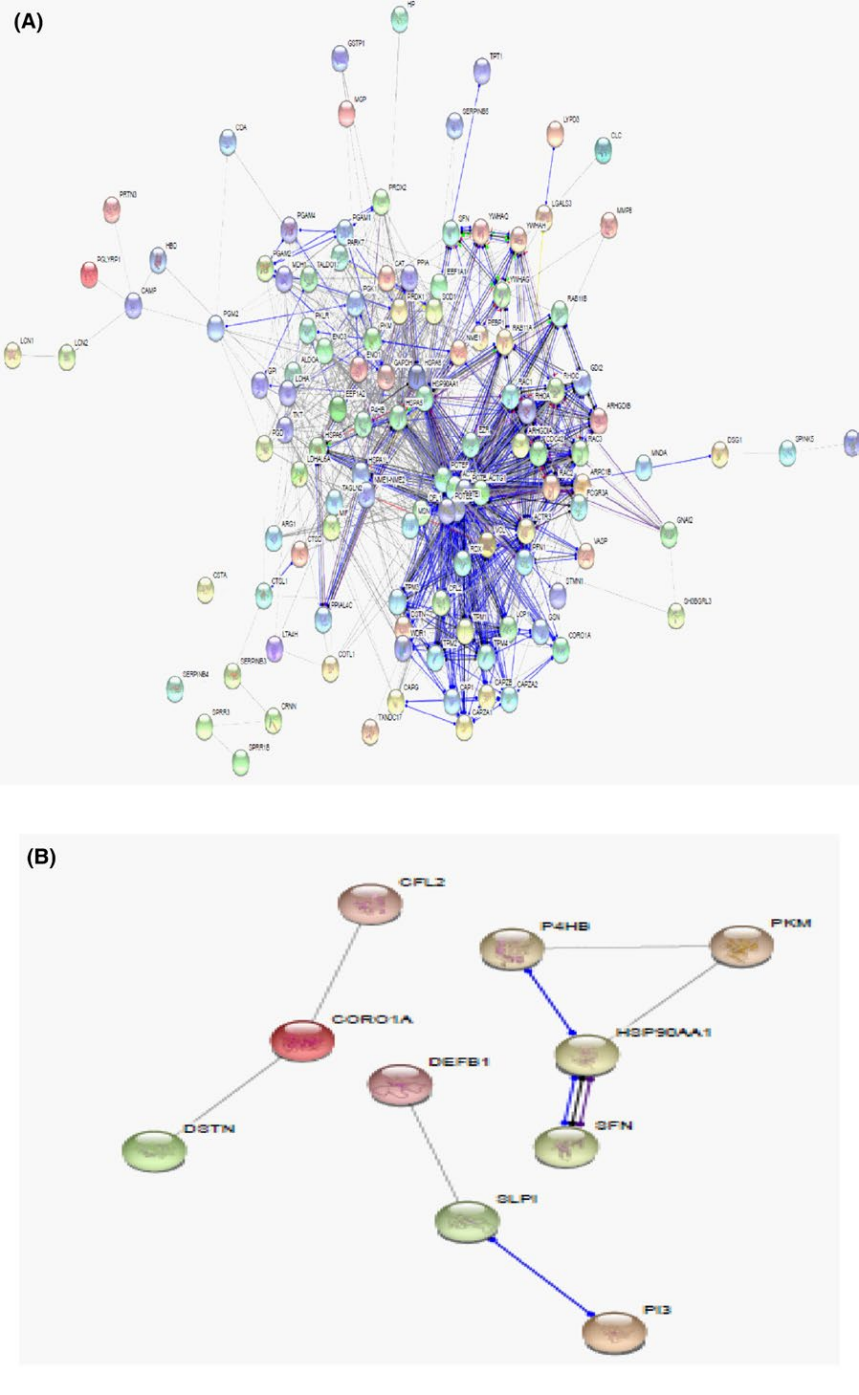
Protein interaction network analysis by STRING. A). Of 132 upregulated proteins between breast cancer/healthy subjects (CAB/HS), 119 proteins were found be functionally linked with 1063 edges with protein‐protein interaction (PPI) enrichment *P*‐value of <1.0e‐16 showing significant interaction. B). Of 22 upregulated proteins between ovarian cancer/healthy subjects (CAO/HS), 10 proteins were functionally linked with 8 edges and PPI enrichment p‐value of 0.0328 showing significant interaction

STRING is a metadatabase program that generates a network of protein interactions from high‐throughput experimental data, literature, and predictions based on genomic context analysis. The networks formed by interacting salivary proteins using this program enabled us to identify proteins that are functionally linked indifferent cellular processes and their association in metastasis in breast and ovarian cancers. Of the 132 upregulated proteins between CAB/HS, 119 proteins were found be functionally linked with 1063 edges with protein‐protein interaction (PPI) enrichment *P*‐value of <1.0e‐16 showing significant interaction. Similarly, of the 22 upregulated proteins between CAO/HS, 10 proteins were functionally linked with 8 edges and PPI enrichment p‐value of 0.0328 showing significant interaction. Among functionally linked proteins within CAB, the majority of the proteins (eg, coronin‐1A, translationally controlled tumor proteins, vasodilator‐stimulated phosphoprotein) demonstrated strong molecular action due to a high score of ≥0.4. In contrast, fewer proteins (eg, coronin 1A, hsp‐90α) were observed to be functionally linked and to interact within the CAO group (Figure 5A,B).

### 3.7 Validation of proteomics results by western blot analysis

**Figure 6:**
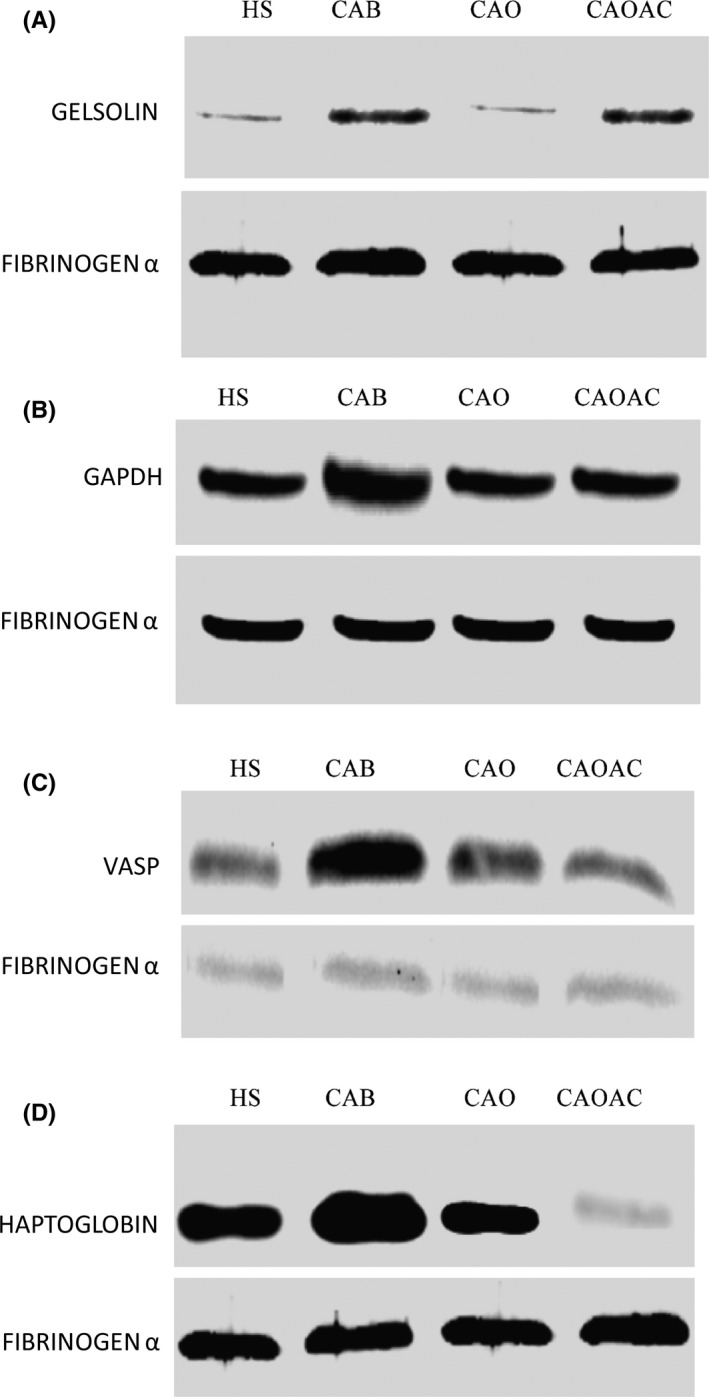
Verification of selected human salivary proteins by western blot. Immunoblots of pooled saliva proteins from healthy subjects (HS), breast cancer (CAB), ovarian cancer (CAO), and ovarian cancer subjects after chemotherapy (CAOAC) patients' groups. (A) Gelsolin, (B) glyceraldehyde‐3‐phosphate dehydrogenase, (C) vasodilator‐stimulated phosphoprotein, and (D) haptoglobin. Western blot analysis verifies their significant upregulation in the CAB patient group compared to other HS, CAO and CAOAC patient groups. Fibrinogen‐α was used as a loading control

Candidate salivary proteins were selected to confirm and quantify their differential expression in all four groups based on protein log_2_ fold changes based on their association with the either breast or ovarian cancer. Western blot (WB) analysis was performed on gelsolin (polyclonal, 1:1000), glyceraldehyde‐3‐phosphate dehydrogenase (1:1000), vasodilator‐stimulated phosphoprotein (1:500), and haptoglobin (1:1000), while fibrinogen‐α was used as the loading control. Gelsolin expression was significantly upregulated in CAB patients compared to HS and CAO patients, while it was downregulated in CAO compared to CAOAC patients. In contrast, no change in abundance was observed in CAO patients compared to HS. Vasodilator‐stimulated phosphoprotein expression was higher in abundance in CAB and CAO patients compared to HS. Similarly, higher abundance of vasodilator‐stimulated phosphoprotein was also observed in CAO compared to CAOAC patients. Haptoglobin and glyceraldehyde‐3‐phosphate dehydrogenase was significantly upregulated in CAB patients compared to all other patient groups (Figure 6A‐D). WB analysis performed on individual samples (N = 5), previously used for the discovery dataset, showed similar expression trends verifying no individual samples influencing the overall differential expression of proteins in pooled samples. Similarly, WB performed on individual samples from an independent cohort (N = 5) had no outliers, and were consistent in their expression. However, haptoglobin had no significant expression in CAOAC patients, and when pooled were downregulated compared to healthy, breast, and ovarian cancer samples, as shown in Supplementary Figure S1A‐D.

## 4 DISCUSSION

Saliva, as a substitute for blood, has been increasing in use as an indicator of patients’ health status and intervention outcomes, including its use as a source of potential markers for early disease diagnosis.^31^ An important aspect that has been relatively unexplored is the use of potential markers to better understand metastatic organotropic behavior of breast and ovarian cancers, which has an incidence rate of 15%‐30%.^3^ Currently, BRCA1/2 mutation, PTEN, and p53 analysis are being studied to uncover organotropism including predictive analysis of HRs to investigate the molecular subtype of breast and ovarian cancers that can metastasize to distant organs using immunohistochemical staining or microarrays.^8^, ^14^, ^16^ Although, early prediction of organotropism can decrease the incidence of cancer‐related mortality in women, these clinical methods are invasive and expensive.^32^ Similarly, neoadjuvant chemotherapy of sensitive/resistant proteins has been studied to determine effective treatments. Thus, the focus of our study was to characterize salivary proteins with the aim of identifying an expression pattern that correlates to organotropic behavior of breast and ovarian cancers, as well as neoadjuvant chemotherapy treatment in ovarian cancer patients.

A comparative analysis of salivary proteins identified in our study was performed with previously reported proteomic studies of breast cancer. For example, Streckfus et al^33^ analysed saliva from breast cancer stage IIa and IIb patients, resulting in the identification of 178 proteins, of which 158 were differentially expressed. In contrast, our study reports identification of 409 proteins from breast cancer patients in stage IV, of which 59 proteins were differentially expressed. Similarly, Cao et al^34^ identified 464 proteins by isobaric tag for relative and absolute quantification (iTRAQ), of which 125 were differentially expressed. Contrastingly, 645 proteins were confidently identified and quantified, of which 409 proteins met the quantitative criteria of identification of two different peptides for analysis in our study.

Metastatic cancer or distant recurrence is usually preceded by several months to years prior to establishment of diagnosis. Similar to pancreatic cancer, which starts to metastasize early.^35^ De novo breast and ovarian cancer metastases are also diagnosed at an early stage, before primary diagnosis.^12^, ^13^ Thus, to understand the metastatic behavior of breast and ovarian cancers, STRING analysis of upregulated proteins, known to be involved in controlling cell motility, was performed to uncover potential interactions that would regulate their downstream function in promoting metastasis, for instance, cell motility‐related proteins such as coronin‐1A (CORO‐1A), a member of the WD repeat protein family, known to regulate cell signaling and cell migration.^36^ In CAB patients, indirect interaction of CORO‐1A with Rac1 through ARCT3 (Figure 5A) has been known to regulate Rac1 associated functions such as cell migration and invasion in multiple cancers, as well as in activation of c‐Jun N‐terminal kinase (JNK) and JNK‐dependent cell motility.^37^ Similarly, Rho small GTPase family proteins (eg, Rab11A, 11B), reorganize actin proteins related to cell motility, and have previously been reported to interact with moesin resulting in collective cell migration.^38^ Moreover, proteins involved in cytoskeletal function or extracellular matrix (ECM) remodeling process, such as VASP, CORO‐1A, CAP1, TPM4, PFN1, and COTL1 which are essential for tumor cell invasion,^39^ were observed to be interconnected with each other or other cell motility proteins such as cofilin, gelsolin, ezrin (Figure 5A). Contrastingly in CAO patients, no such interaction was observed (Figure 5B). Other cell motility‐related proteins like actin‐cytoskeletal modulating proteins including ezrin‐redixin‐moesin family proteins,^40^ F‐actin‐capping proteins,^41^ cell division control protein‐42, and Rab and Rho family proteins^42^ were functionally linked (Figure 5A), but were either downregulated or showed basal expression in CAO subjects. These proteins with multiple functions could play a significant role, as reported in previous studies, in metastasis through assisting in the assembly or disassembly of actin, microtubules and cytoskeleton proteins of cells. Of significant interest, several estrogen responsive proteins such as HSP 70 and 90 were upregulated in CAB patients and have been previously linked to regulation of transcriptional activity.^43^

In addition to 409 identified proteins, 352 proteins were common in all groups, while 57 were either present/absent exclusively in one group or common in any two/three groups. For example, hepatoma‐derived growth factor (HDGF), a heparin binding protein, identified only in CAB patients has previously been reported to have a putative role in carcinogenesis and metastatic invasiveness of breast cancer through epithelial‐mesenchymal transition (EMT).^44^ Similarly, gelsolin, a tumor suppressor protein, was upregulated in CAB patients, while it is downregulated in CAO subjects. The change in abundance of gelsolin in CAB follows the same trend that has previously been reported in breast cancer and associated with the reversion of the transformed phenotype in malignant cells.^45^ Additionally, upregulation of gelsolin has also been reported in erbB‐2+ and EGFR+ as well as in aggressive breast cancer phenotype cell lines, resulting in enhanced motility, invasion, and metastatic potential.^45^ Notably, upregulated expression of gelsolin in CAB patients is not surprising since study subjects were a confirmed population subset with erbB‐2+ and EGFR+.^45^ In contrast, downregulation of gelsolin in CAO subjects confirms previous results which also reported its lower expression in highly aggressive and poorly differentiated carcinomas.^46^

Another family of proteins, the high‐mobility group proteins (HMGPB1 and HMGPB2), also known as amphoterin, was detected in CAB, CAO, and CAOAC subjects while it was absent in HS. Brezniceanu et al^47^ confirmed high expression of HMGPB1 in patients with breast carcinomas and a lack of HMGPB1 expression in healthy breast tissue.^47^ High‐serum HMGPB1 have also been associated with advanced stage and lymph node metastasis in ovarian cancer, suggesting a crucial role in ovarian cancer development.^48^ Downregulation of HMGPB1 in CAOAC patients was consistent with a previously reported study showing lower expression in post‐chemotherapy subjects due to their resistance to neoadjuvant drugs,^49^ possibly suggesting their prognostic role in response to paclitaxel and carboplatin drugs. In addition, upregulation of HSP90ɑ and translationally controlled tumor protein (TPT‐1) in both CAB and CAO patients, and upregulation of HSP70 in CAO patients compared to HS, is consistent with previous studies which reported their association with enhanced tumor invasiveness and malignancy.^50^, ^51^ In contrast, HSP70, HSP90α, and TPT‐1 were not detected in CAOAC subjects, possibly suggesting their chemosensitivity toward paclitaxel and carboplatin drug therapy.

Other oncoproteins previously identified were known to be involved in progression and development of various cancerous tissues such as esophageal squamous cell carcinoma (ESCC), and breast and ovarian cancers.^52^, ^53^, ^54^ For example, suprabasin (SBSN), showed no change in abundance in CAB and CAO subjects but was upregulated in CAOAC patients, which may be in response to neoadjuvant paclitaxel and carboplatin drugs. Similarly, stathmin (STMN1), also known as Op18, is known to be involved in cell invasion and cancer metastasis,^52^ and was observed to be lower in abundance in CAO subjects. However, it is at odds with previous reports, whereby upregulation of STMN1in breast cancer cell lines promote cell proliferation and carcinogenesis.^53^ This difference in abundance between CAB and CAO subjects could be due to cell or tissue type and specific to transformation.^54^ Not surprisingly, STMN1 was not detected in CAOAC subjects, suggesting its possible sensitivity toward the neoadjuvant drugs, paclitaxel^55^ and carboplatin. Of note, VASP and coronin‐1A proteins, known to be involved in the cell migration process through ECM modulation,^39^ was upregulated in CAO patients, but had lower expression in CAOAC patients, possibly in response to neoadjuvant drug therapy. VASP has also been previously reported to be functionally regulated by PKG1α and involved in chemoresistance, cell migration and invasion in ovarian cancer.^56^

The comparison between ovarian cancer and drug‐treated ovarian cancer patients was performed to identify differentially expressed proteins that could serve as markers to monitor progression of the therapeutic influence of neoadjuvant drugs. For example, Suprabasin is involved in progression and development of breast, ovarian and esophageal squamous cell carcinoma (ESCC).^52^, ^53^, ^54^ Consistent with previous reports, suprabasin was upregulated in drug‐treated samples but showed no change in expression in non‐treated samples, and likely linked to the effects of neoadjuvant drugs. Similarly, prosaposin, an antimetastatic protein, inhibits the migration of ovarian cancer via stimulation of p53, was upregulated in drug‐treated samples, compared to untreated samples, and could be associated with the drug sensitivity.^57^ Metastasis promoting (by targeting the ECM substrate) proteins like MMP‐9 and collagenase were upregulated in treated samples compared to untreated samples, consistent with previous studies reporting enhanced activity of these proteins, and associated with drug resistance.^58^ Furthermore, a small proline‐rich protein, previously reported to be involved in promoting ovarian cancer,^59^ was significantly upregulated in drug‐treated samples as compared to non‐treated samples, and possibly associated with drug resistance. Taken together, significant changes in the expression of proteins in treated and non‐treated samples could be related to sensitivity or resistance toward drugs used for the treatment.

In summary, this is the first proteomic study to identify a pattern of differentially expressed salivary proteins as indicators of metastatic organotropism potential of breast and ovarian cancers, as well as their response to neoadjuvant (paclitaxel and carboplatin) drug therapy. Our study using whole saliva identified a total of 646 proteins, of which 409 proteins were identified, including 57 proteins which were either present/absent exclusively in one group or common in any of the two/three groups. Most metastatic‐related proteins (eg, coronin‐1A, TPT‐1, VASP, HSP90α, cofilin, and Arp 2/3) were identified in both CAB and CAO patients, suggesting their association with organotropism of metastatic breast and ovarian cancers. Besides, protein interaction analysis revealed the majority of upregulated proteins in CAB patients were functionally linked, possibly signifying their aggressiveness of metastasis of breast cancer than ovarian cancer. Similarly, CORO‐1A and VASP were downregulated, and SBSN, MMP‐9 prosaposin, and small proline rich protein were upregulated. The expression of STMN1 was lower than the detectable limit, possibly in response to combined drug therapy, suggesting their possible association with a chemosensitive/resistant response. Considering the scarcity of literature available on organotropism to date, rigorous research is required using a large cohort of patients, with multiple cancers to confidently identify the proteins that may act as potential biomarkers and to better understand the complexity behind organotropism.

## 5 SUPPLEMENTARY MATERIAL

Label‐free quantitative analysis data excel file. Table S1, contain total proteins identified in four groups and their CV%. Tables [Link], [Link], [Link], [Link] contains list of proteins which showed changes in abundance between CAB vs HS, CAO vs HS, CAB vs CAO, and CAO vs. CAOAC patients, respectively. Table S6 contains values obtained by a one‐way ANOVA for all four sample groups. Supplementary Figure S1A‐D shows western blot analysis across four groups (HS, CAB, CAO, and CAOAC) on individual samples (cohort 1: subjects/patients (N = 5) used for the discovery dataset and cohort 2: subjects/patient (N = 5), an independent cohort).

## CONFLICT OF INTEREST

The authors declare no conflict of interest.

## AUTHOR CONTRIBUTIONS

K.G. designed, performed experiments, and analyzed data. A.M. performed and interpreted histology and mammography, CT‐Scan and FDG‐PET reports. K.A. conceived, designed, interpreted data, and supervised the study. K.G. and K.A. wrote the manuscript.

## References

[1] Ernst & Young . Call for Action: Expanding cancer care in India. https://www.ey.com/Publication/vwLUAssets/ey-expanding-cancer-care-for-women-in-india-formatted-sep-19-500-pm-lowrez/$File/ey-expanding-cancer-care-for-women-in-india-formatted-sep-19-500-pm-lowrez.pdf Accessed April 4, 2018.

[2] Ferlay J , Soerjomataram I , Dikshit R , et al. Cancer incidence and mortality worldwide: sources, methods and major patterns in GLOBOCAN 2012. Int J Cancer. 2015;136:E359–E386. 25220842 10.1002/ijc.29210

[3] Voogd AC , Nielsen M , Peterse JL , et al; Danish Breast Cancer Cooperative Group, Breast Cancer Cooperative Group of the European Organization for Research and Treatment of Cancer . Differences in risk factors for local and distant recurrence after breast‐conserving therapy or mastectomy for stage I and II breast cancer: pooled results of two large european randomized trials. J Clin Oncol. 2001;19:1688‐1697. 11250998 10.1200/JCO.2001.19.6.1688

[4] Torre LA , Islami F , Siegel RL , Ward EM , Jemal A . Global Cancer in Women: Burden and Trends. Cancer Epidemiol Biomark Prev. 2017;26:444‐457. 10.1158/1055-9965.EPI-16-0858 28223433

[5] Longacre M , Snyder NA , Housman G , et al. A Comparative analysis of genetic and epigenetic events of breast and ovarian cancer related to tumorigenesis. Int J Mol Sci. 2016;17:759. 10.3390/ijms17050759 PMC4881580 27213343

[6] Sundar S , Khetrapal‐Singh P , Frampton J , et al. Harnessing genomics to improve outcomes for women with cancer in India: key priorities for research. Lancet Oncol. 2018;19:e102‐e112. 29413464 10.1016/S1470-2045(17)30726-X

[7] Lambert AW , Pattabiraman DR , Weinberg RA . Emerging biological principles of metastasis. Cell. 2017;168:670‐691. 28187288 10.1016/j.cell.2016.11.037 PMC5308465

[8] Chen W , Hoffmann AD , Liu H , Liu X . Organotropism: new insights into molecular mechanisms of breast cancer metastasis. NPJ Precis Oncol. 2018;2:4. 29872722 10.1038/s41698-018-0047-0 PMC5871901

[9] Akhter MZ , Sharawat SK , Kumar V , et al. Aggressive serous epithelial ovarian cancer is potentially propagated by EpCAM+CD45+ phenotype. Oncogene. 2018;37:2089‐2103. 29379166 10.1038/s41388-017-0106-y

[10] Bigorie V , Morice P , Duvillard P , et al. Ovarian metastases from breast cancer. Cancer. 2010;116:799‐804. 20041486 10.1002/cncr.24807

[11] Klein RL , Brown AR , Gomez‐Castro CM , et al. Ovarian cancer metastatic to the breast presenting as inflammatory breast cancer: a case report and literature review. Journal of Cancer. 2010;1:27‐31. 20842221 10.7150/jca.1.27 PMC2931350

[12] Howlader N , Cronin KA , Kurian AW , Andridge R . Differences in breast cancer survival by molecular subtypes in the United States. Cancer Epidemiol Biomark Prev. 2018;27:619‐626. 10.1158/1055-9965.EPI-17-0627 29593010

[13] Koshiyama M , Matsumura N , Konishi I . Subtypes of ovarian cancer and ovarian cancer screening. Diagnostics. 2017;7:12. 10.3390/diagnostics7010012 PMC5373021 28257098

[14] Çelik A , Acar M , Moroski Erkul C , Gunduz E , Gunduz M . Relationship of breast cancer with ovarian cancer 2015 In A concise review of molecular pathology of breast cancer, Mehmet Gunduz, IntechOpen. https://www.intechopen.com/books/a-concise-review-of-molecular-pathology-of-breast-cancer/relationship-of-breast-cancer-with-ovarian-cancer. Accessed July 29, 2018.

[15] Schrijver W , van Diest PJ , and Moelans CB ; Consortium, D. D. B. C. M . Unravelling site‐specific breast cancer metastasis: a microRNA expression profiling study. Oncotarget. 2017;8:3111‐3123. 27902972 10.18632/oncotarget.13623 PMC5356868

[16] Wooster R , Weber BL . Breast and ovarian cancer. N Engl J Med. 2003;348:2339‐2347. 12788999 10.1056/NEJMra012284

[17] Skaane P . Studies comparing screen‐film mammography and full‐field digital mammography in breast cancer screening: Updated review. Acta Radiologica (Stockholm, Sweden : 1987). 2009;50:3‐14. 10.1080/02841850802563269 19037825

[18] Nossov V , Amneus M , Su F , et al. The early detection of ovarian cancer: from traditional methods to proteomics. Can we really do better than serum CA‐125? Am J Obstet Gynecol. 2008;199:215‐223. 18468571 10.1016/j.ajog.2008.04.009

[19] Szabo C , Masiello A , Ryan JF , Brody LC . The breast cancer information core: database design, structure, and scope. Hum Mutat. 2000;16:123‐131. 10923033 10.1002/1098-1004(200008)16:2<123::AID-HUMU4>3.0.CO;2-Y

[20] Agha‐Hosseini F , Mirzaii‐Dizgah I , Rahimi A . Correlation of serum and salivary CA15‐3 levels in patients with breast cancer. Med Oral Patol Oral Cir Bucal. 2009;14:e521‐e524. 19680209 10.4317/medoral.14.e521

[21] Bocheva Y , Bochev P , Ivanov S . Ca‐125 in diagnosis and monitoring of patients with ovarian cancer. Akush Ginekol (Sofiia). 2015;54:11‐17. 25909124

[22] Bingle L , Singleton V , Bingle CD . The putative ovarian tumour marker gene HE4 (WFDC2), is expressed in normal tissues and undergoes complex alternative splicing to yield multiple protein isoforms. Oncogene. 2002;21:2768‐2773. 11965550 10.1038/sj.onc.1205363

[23] Rapado‐González Ó , Majem B , Muinelo‐Romay L , López‐López R , Suarez‐Cunqueiro M . Cancer salivary biomarkers for tumours distant to the oral cavity. Int J Mol Sci. 2016;17:1531. 10.3390/ijms17091531 PMC5037806 27626410

[24] Podzimek S , Vondrackova L , Duskova J , Janatova T , Broukal Z . Salivary markers for periodontal and general diseases. Dis Markers. 2016;2016: 1‐8. 10.1155/2016/9179632 PMC4837271 27143814

[25] Ambatipudi KS , Swatkoski S , Moresco JJ , et al. Quantitative proteomics of parotid saliva in primary Sjögren's syndrome. Proteomics. 2012;12:3113‐3120. 22888089 10.1002/pmic.201200208 PMC3806318

[26] Lau CS , Wong D . Breast cancer exosome‐like microvesicles and salivary gland cells interplay alters salivary gland cell‐derived exosome‐like microvesicles in vitro. PLoS ONE. 2012;7:e33037. 22448232 10.1371/journal.pone.0033037 PMC3308964

[27] Babicki S , Arndt D , Marcu A , et al. Heatmapper: web‐enabled heat mapping for all. Nucleic Acids Res. 2016;44:W147–W153. 27190236 10.1093/nar/gkw419 PMC4987948

[28] Mi H , Muruganujan A , Thomas PD . PANTHER in 2013: modeling the evolution of gene function, and other gene attributes, in the context of phylogenetic trees. Nucleic Acids Res. 2013;41:D377‐D386. 23193289 10.1093/nar/gks1118 PMC3531194

[29] Zheng WQ , Lu J , Zheng JM , Hu FX , Ni CR . Variation of ER status between primary and metastatic breast cancer and relationship to p53 expression*. Steroids. 2001;66:905‐910. 11711119 10.1016/s0039-128x(01)00121-0

[30] Pourzand A , Fakhree M , Hashemzadeh S , Halimi M , Daryani A . Hormone receptor status in breast cancer and its relation to age and other prognostic factors. Breast Cancer (Auckl). 2011;5: 87‐92. 21695095 10.4137/BCBCR.S7199 PMC3117624

[31] Hu S , Loo JA , Wong DT . Human saliva proteome analysis and disease biomarker discovery. Expert Rev Proteomics. 2007;4:531‐538. 17705710 10.1586/14789450.4.4.531

[32] Mallath MK , Taylor DG , Badwe RA , et al. The growing burden of cancer in India: epidemiology and social context. Lancet Oncol. 2014;15:e205–e212. 24731885 10.1016/S1470-2045(14)70115-9

[33] Streckfus CF , Storthz KA , Bigler L , Dubinsky WP . A comparison of the proteomic expression in pooled saliva specimens from individuals diagnosed with ductal carcinoma of the breast with and without lymph node involvement. J Oncol. 2009;2009:737619. 20052393 10.1155/2009/737619 PMC2801014

[34] Cao M‐Q , Wu Z‐Z , Wu W‐K . Identification of salivary biomarkers in breast cancer patients with thick white or thick yellow tongue fur using isobaric tags for relative and absolute quantitative proteomics.. Zhong Xi Yi Jie He Xue Bao. 2011;9:275‐280. 21419079 10.3736/jcim20110307

[35] Ryan DP , Hong TS , Bardeesy N . Pancreatic Adenocarcinoma. N Engl J Med. 2014;371:2139‐2141. 25427123 10.1056/NEJMc1412266

[36] Rybakin V , Clemen CS . Coronin proteins as multifunctional regulators of the cytoskeleton and membrane trafficking. BioEssays. 2005;27:625‐632. 15892111 10.1002/bies.20235

[37] Liu S , Yu M , He Y , et al. Melittin prevents liver cancer cell metastasis through inhibition of the Rac1‐dependent pathway. Hepatology. 2008;47:1964‐1973. 18506888 10.1002/hep.22240

[38] Emery G , Ramel D . Cell coordination of collective migration by Rab11 and Moesin. Commun Integr Biol. 2013;6:e24587. 23956813 10.4161/cib.24587 PMC3737754

[39] Pitteri SJ , Kelly‐Spratt KS , Gurley KE , et al. Tumor microenvironment‐derived proteins dominate the plasma proteome response during breast cancer induction and progression. Can Res. 2011;71:5090‐5100. 10.1158/0008-5472.CAN-11-0568 PMC3148311 21653680

[40] Lan M , Kojima T , Murata M , et al. Phosphorylation of ezrin enhances microvillus length via a p38 MAP‐kinase pathway in an immortalized mouse hepatic cell line. Exp Cell Res. 2006;312:111‐120. 16274688 10.1016/j.yexcr.2005.09.018

[41] Croucher DR , Rickwood D , Tactacan CM , Musgrove EA , Daly RJ . Cortactin modulates RhoA activation and expression of Cip/Kip cyclin‐dependent kinase inhibitors to promote cell cycle progression in 11q13‐amplified head and neck squamous cell carcinoma cells. Mol Cell Biol. 2010;30:5057‐5070. 20805359 10.1128/MCB.00249-10 PMC2953065

[42] Porter AP , Papaioannou A , Malliri A . Deregulation of Rho GTPases in cancer. Small GTPases. 2016;7:123‐138. 27104658 10.1080/21541248.2016.1173767 PMC5003542

[43] Zhu Z , Boobis AR , Edwards RJ . Identification of estrogen‐responsive proteins in MCF‐7 human breast cancer cells using label‐free quantitative proteomics. Proteomics. 2008;8:1987‐2005. 18491314 10.1002/pmic.200700901

[44] Chen S‐C , Kung M‐L , Hu T‐H , et al. Hepatoma‐derived growth factor regulates breast cancer cell invasion by modulating epithelial–mesenchymal transition. J Pathol. 2012;228:158‐169. 22247069 10.1002/path.3988

[45] Thor AD , Edgerton SM , Liu S , Moore DH , Kwiatkowski DJ . Gelsolin as a negative prognostic factor and effector of motility in erbB‐2‐positive epidermal growth factor receptor‐positive breast cancers. Clin Cancer Res. 2001;7:2415‐2424. 11489821

[46] Noske A , Denkert C , Schober H , et al. Loss of Gelsolin expression in human ovarian carcinomas. Eur J Cancer. 2005;41:461‐469. 15691647 10.1016/j.ejca.2004.10.025

[47] Brezniceanu M‐L , Völp K , Bösser S , et al. HMGB1 inhibits cell death in yeast and mammalian cells and is abundantly expressed in human breast carcinoma. FASEB J. 2003;17:1295‐1297. 12759333 10.1096/fj.02-0621fje

[48] Wang H , Li Z , Sun Y , et al. Relationship between high‐mobility group box 1 overexpression in ovarian cancer tissue and serum: a meta‐analysis. Onco Targets Ther. 2015;8:3523‐3531. 26664135 10.2147/OTT.S93357 PMC4669932

[49] Bernardini M , Lee C‐H , Beheshti B , et al. High‐resolution mapping of genomic imbalance and identification of gene expression profiles associated with differential chemotherapy response in serous epithelial ovarian cancer. Neoplasia (New York, N.Y.). 2005;7:603‐613. 10.1593/neo.04760 PMC1501280 16036111

[50] Wang X , Song X , Zhuo W , et al. The regulatory mechanism of Hsp90α secretion and its function in tumor malignancy. Proc Natl Acad Sci. 2009;106:21288‐21293. 19965370 10.1073/pnas.0908151106 PMC2795546

[51] Nagano‐Ito M , Ichikawa S . Biological effects of Mammalian translationally controlled tumor protein (TCTP) on cell death, proliferation, and tumorigenesis. Biochem Res Int. 2012;2012:204960. 22675633 10.1155/2012/204960 PMC3364544

[52] Kouzu Y , Uzawa K , Koike H , et al. Overexpression of stathmin in oral squamous‐cell carcinoma: correlation with tumour progression and poor prognosis. Br J Cancer. 2006;94:717‐723. 16495930 10.1038/sj.bjc.6602991 PMC2361217

[53] Bièche I , Lachkar S , Becette V , et al. Overexpression of the stathmin gene in a subset of human breast cancer. Br J Cancer. 1998;78:701‐709. 9743287 10.1038/bjc.1998.565 PMC2062973

[54] Price DK , Ball JR , Bahrani‐Mostafavi Z , et al. The phosphoprotein Op18/stathmin is differentially expressed in ovarian cancer. Cancer Invest. 2000;18:722‐730. 11107442 10.3109/07357900009012204

[55] Balachandran R , Welsh MJ , Day BW . Altered levels and regulation of stathmin in paclitaxel‐resistant ovarian cancer cells. Oncogene. 2003;22:8924‐8930. 14654788 10.1038/sj.onc.1207060

[56] Wong Janica C , Fiscus RR .Protein Kinase G‐Iα Hyperactivation and VASP phosphorylation in promoting ovarian cancer cell migration and platinum resistance In: Díaz-Padilla I, ed. Ovarian cancer – a clinical and translational update. Rijeka, Croatia: Intech Open Access Publisher; 2013:251-273.

[57] Wang S , Blois A , El Rayes T , et al. Development of a prosaposin‐derived therapeutic cyclic peptide that targets ovarian cancer via the tumor microenvironment. Sci Transl Med. 2016;8:329ra34. 10.1126/scitranslmed.aad5653 PMC6261358 26962158

[58] Al‐Batran S , Wirtz RM , Pauligk C , et al. Association of elevated matrix metalloproteinase‐9 (MMP‐9) mRNA expression levels with resistance to chemotherapy and survival in patients with metastatic gastric cancer receiving first‐line chemotherapy: results from the FLO versus FLP gastric cancer phase III trial of the AIO. J Clin Oncol. 2008;26:4544‐4544.18824706

[59] Chudasama D , Bo V , Hall M , et al. Identification of cancer biomarkers of prognostic value using specific gene regulatory networks (GRN): a novel role of RAD51AP1 for ovarian and lung cancers. Carcinogenesis. 2018;39:407‐417. 29126163 10.1093/carcin/bgx122 PMC5862298

