# Supplementary figures and images for: In search of the altering salivary proteome in metastatic breast and ovarian cancers
Source: FASEB Bioadv. 2019 Jan 30;1(3):191–207. doi: 10.1096/fba.2018-00029 (PMC6996400; doi:10.1096/fba.2018-00029)

Figure 1A

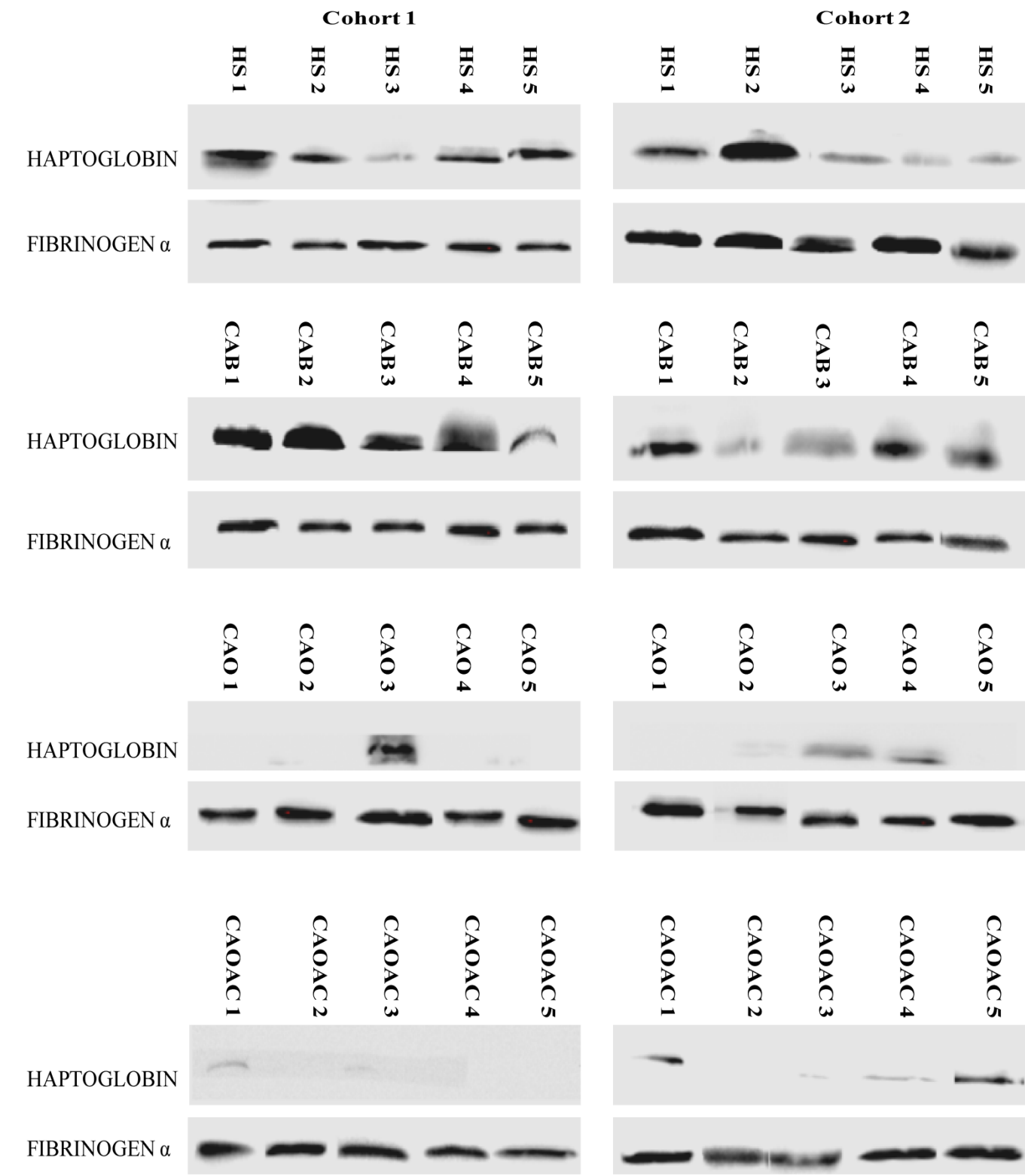

Figure 1B

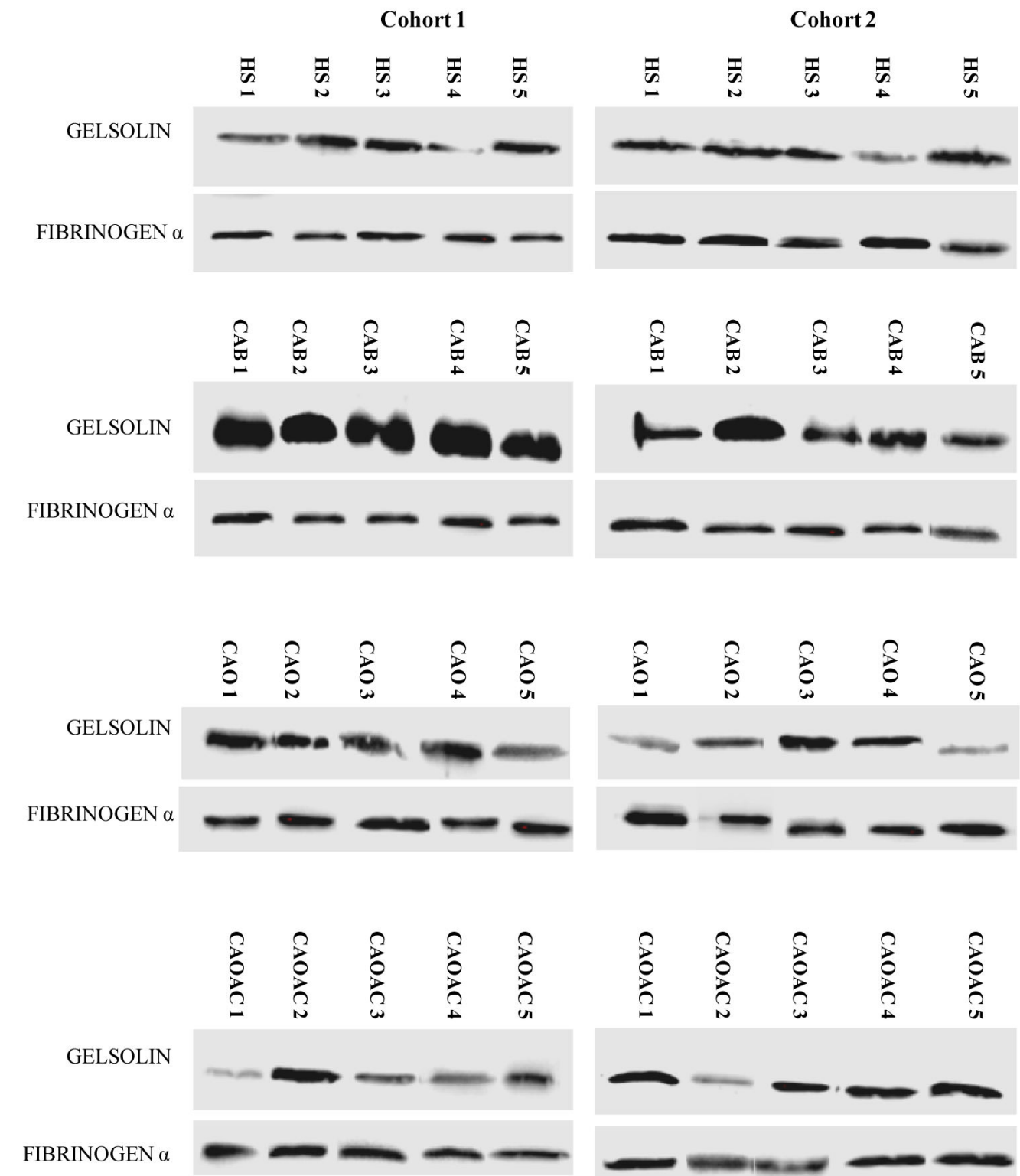

Figure 1C

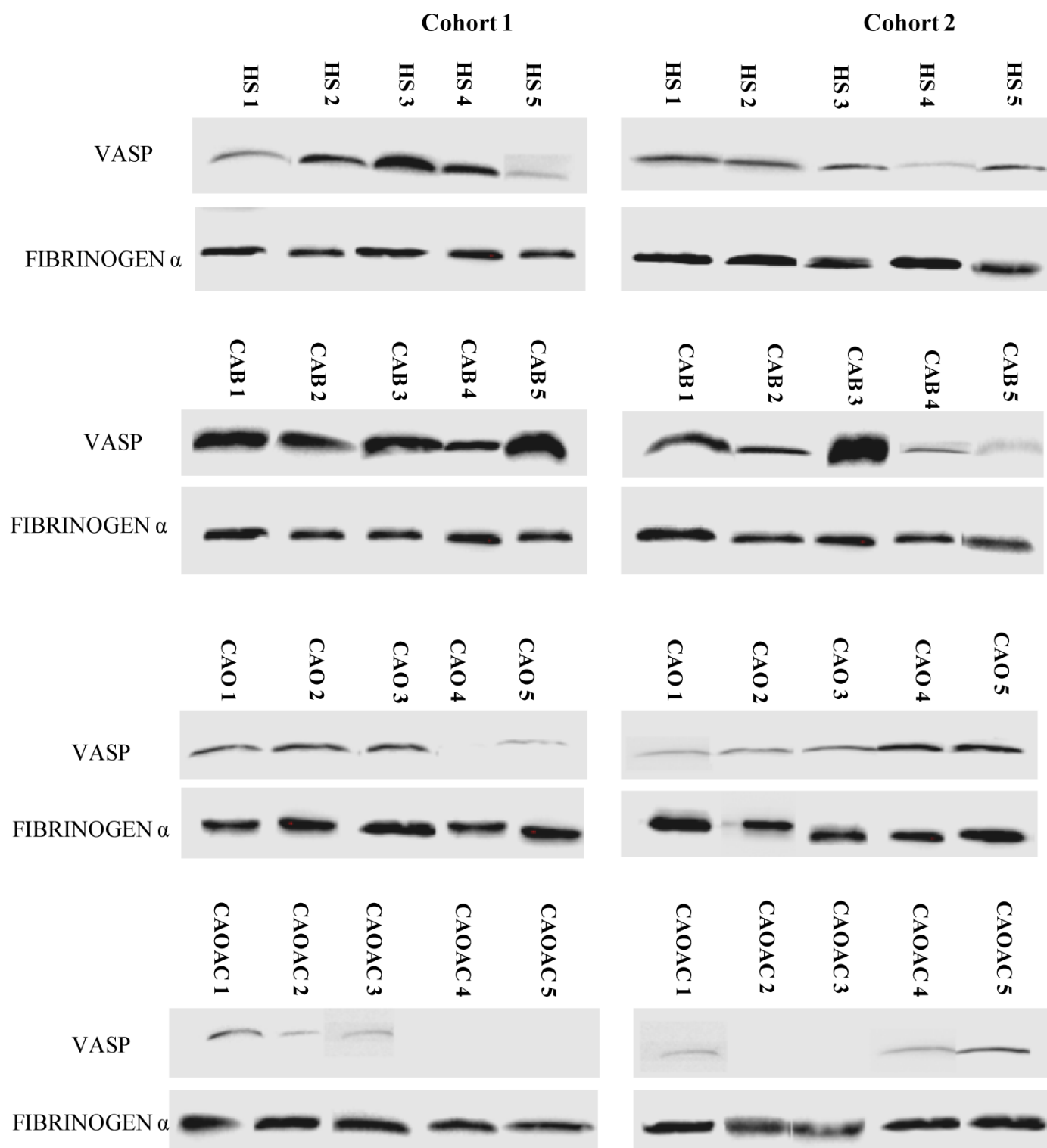

Figure 1D

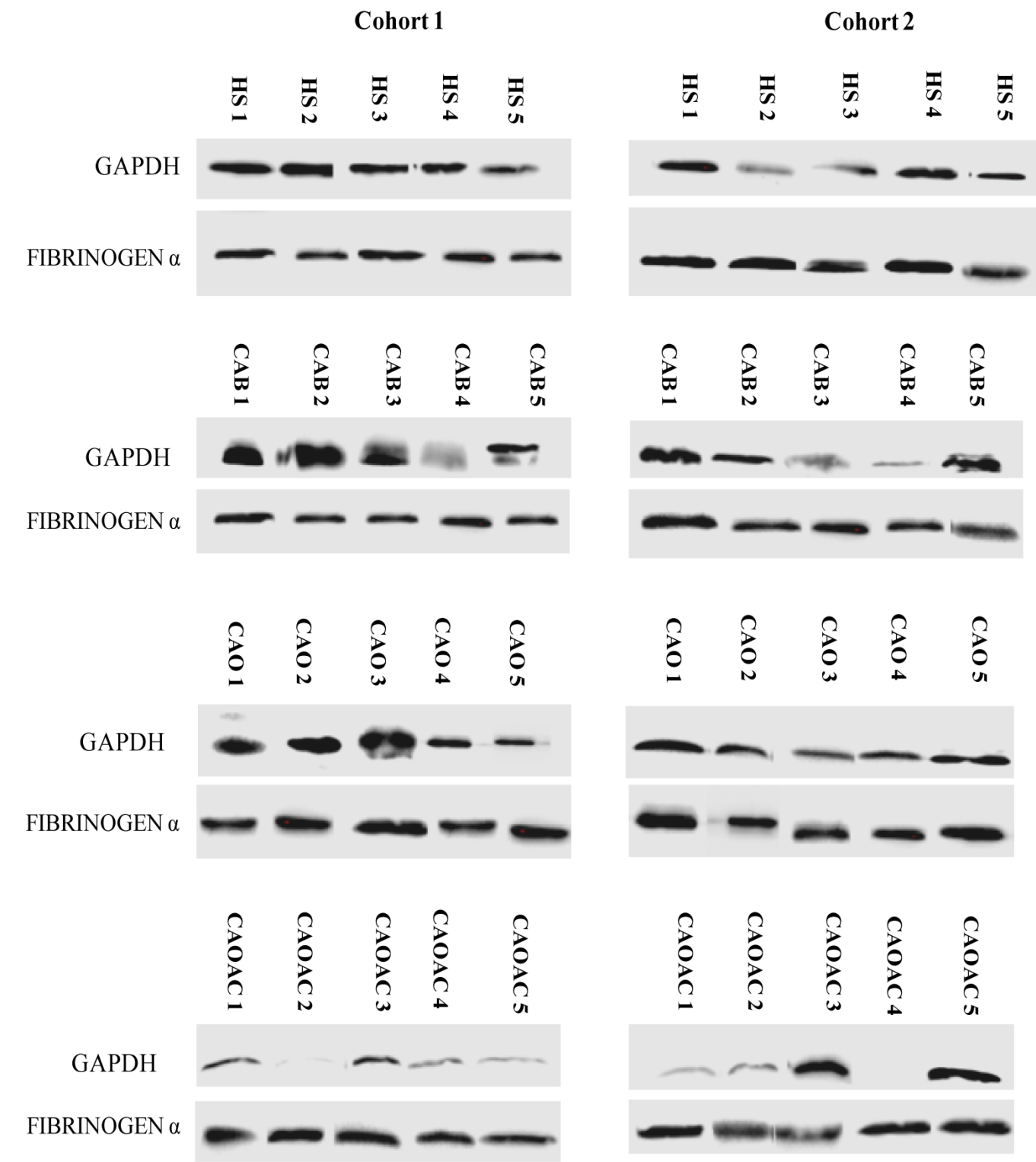

Supplement: Supplementary file 1 [file FBA2-1-191-s001.pdf]
